# *Candida auris* in the Middle East and North Africa: From Emerging Threat to Regional Healthcare Challenge

**DOI:** 10.3390/medsci14060679

**Published:** 2026-10-17

**Authors:** Bahgat Fayed

**Affiliations:** Department of Chemistry of Natural and Microbial Products, Pharmaceutical and Drug Industries Research Institute, National Research Centre, 33 El Bohouth St., Dokki, Giza 12622, Egypt; bahgatfayed1982@yahoo.com; Tel.: +20-1098292083

**Keywords:** *Candida auris*, infection, COVID-19, resistance

## Abstract

*Candida auris* (also termed *Candidozyma auris*) (*C. auris*) is an emerging healthcare-associated yeast that has become a major concern because of its persistence in hospitals, frequent misidentification, multidrug resistance, and ability to cause outbreaks in critically ill patients. This review synthesizes the available evidence from the Gulf region, the Eastern Mediterranean region, and North Africa, with emphasis on reported case patterns, epidemiologic expansion, clade distribution, antifungal resistance, colonization-to-infection transition, mortality, and the impact of the COVID-19 period. The Gulf region carries the highest documented burden, with recurrent hospital outbreaks and large surveillance series reported from Saudi Arabia, Qatar, the United Arab Emirates, Kuwait, Oman, and Bahrain. Across the Eastern Mediterranean region, emergence has been more heterogeneous, but major amplification was observed during the COVID-19 era, particularly in Lebanon and Israel. North African data remain more limited, with Egypt and Algeria representing the main published settings; nevertheless, the available findings indicate recent emergence rather than true absence. Across the region, clade I predominates in most countries, suggesting a largely homogeneous South Asian lineage with efficient healthcare-associated transmission. Fluconazole resistance is nearly universal, amphotericin B susceptibility is variable, and echinocandin activity is usually preserved, although resistance to all major antifungal classes has been documented. Colonization often precedes invasive infection, especially among patients with ICU exposure, invasive devices, and prolonged hospitalization. Mortality remains substantial and is highest among patients with bloodstream infection or progression from colonization to invasive disease. The regional pattern is likely driven by a combination of healthcare infrastructure, surveillance intensity, diagnostic capacity, outbreak control, and pandemic-related strain on infection prevention systems.

## 1. Introduction

*Candida auris* (also termed *Candidozyma auris*) (*C. auris*) is an emerging, healthcare-associated yeast [1]. It has become a major concern for clinical microbiology and infection prevention because of its ability to persist in hospital environments, colonize patients for prolonged periods, spread efficiently within wards, and evade routine laboratory identification [2]. The pathogen is typically recovered from critically ill or immunocompromised patients and may cause bloodstream infection, urinary infection, wound infection, otologic disease, and other invasive syndromes [3,4]. Further, *C. auris* forms adherent biofilms on medical devices and host surfaces, contributing to antifungal tolerance and persistence [4,5]. Although structurally simpler than those of *Candida albicans (C. albicans)*, these biofilms exhibit reduced susceptibility to azoles and amphotericin B due to efflux activity and extracellular matrix effects [6]. Biofilm-associated dispersal cells are released into the environment and bloodstream, displaying enhanced resistance and stress tolerance, thereby facilitating device-associated infection and nosocomial transmission [7]. The World Health Organization classified *C. auris* among the highest-priority fungal threats in its fungal priority pathogens list, reflecting the need for stronger surveillance, diagnostics, and antifungal stewardship [8].

From an epidemiologic standpoint, *C. auris* is not a single uniform lineage. Whole-genome studies demonstrated at least four major phylogeographic clades, traditionally associated with South Asia, East Asia, Africa, and South America, while a genetically distinct isolate from Iran was proposed as a potential fifth clade [9,10]. This clade structure is relevant not only for evolutionary interpretation but also for regional surveillance, because it suggests independent emergence and localized transmission rather than a single global dissemination event. The recognition of distinct clades also has practical implications for outbreak investigation, resistance profiling, and molecular tracking across hospitals and countries. Further, clinical outcome data indicate that *C. auris* infection can be associated with substantial mortality, especially in patients with severe comorbidity, intensive care exposure, invasive devices, or delayed diagnosis. A systematic review and meta-analysis performed during the COVID-19 era estimated a pooled mortality rate of 37% among patients with *C. auris* infection in the context of COVID-19, underscoring the gravity of co-infection and the fragility of affected patient populations [11]. Earlier reviews also emphasized high fatality, recurrent candidemia, therapeutic failure, and the difficulty of interpreting susceptibility testing because of resistance variability and laboratory misidentification [12]. In practice, mortality is influenced by host factors as much as by fungal virulence, but the organism clearly remains a high-consequence pathogen.

The COVID-19 pandemic amplified the relevance of *C. auris* by increasing the density of critically ill patients, prolonged ICU stays, broad-spectrum antimicrobial exposure, corticosteroid use, and mechanical ventilation, all of which favor invasive fungal disease and horizontal transmission. This effect was especially visible in the Middle East. For instance, in Israel, a nationwide outbreak increased sharply during COVID-19 hospitalizations, and in Lebanon, where the first hospital outbreak was reported during the pandemic, half of the affected patients had COVID-19 before isolation of the yeast. Similar hospital-associated transmission has been documented in Kuwait, Oman, Saudi Arabia, and later in other Middle Eastern settings, indicating that the pandemic did not create the problem but intensified pre-existing transmission pathways and diagnostic pressures [13,14].

The Middle East was selected as the focus of this review. This is because the available evidence is regionally important but fragmented across single-case reports, small hospital series, outbreak investigations, and genomic studies, with marked heterogeneity in surveillance intensity, diagnostic capacity, and publication output between countries. A prior review before the COVID-19 epidemic already highlighted *C. auris* as an emerging pathogen in Middle Eastern countries, but the literature has expanded substantially since then, particularly during and after the COVID-19 era [15]. The novelty of the present review lies in integrating the COVID-19/post-COVID-19 evidence into a single regional framework, with emphasis on epidemiology, clade distribution, clinical impact, mortality, laboratory identification, antifungal resistance, and country-level evidence gaps. Such synthesis is needed to inform surveillance, infection control, and future research priorities in the region.

## 2. Method

A structured literature search was conducted to identify published evidence concerning *C. auris* in the Middle East and North Africa. The search was performed using PubMed/MEDLINE, Scopus, and Web of Science databases. In addition, the reference lists of relevant review articles and primary studies were manually screened to identify potentially relevant publications that may not have been retrieved through the electronic database search. The search strategy combined terms referring to the organism, geographical location, and major clinical and epidemiological characteristics of *C. auris*. The main search terms included “*Candida auris*” OR “*Candidozyma auris*”, combined with terms including “Middle East,” “North Africa,” “outbreak,” “candidemia,” “colonization,” “antifungal resistance,” “clade,” “misidentification,” and “COVID-19.” Country-specific searches were also conducted using the names of countries within the predefined geographical scope. Boolean operators (AND/OR) were used to combine the principal concepts. The search covered publications available from 2010 THROUGH 2026. The geographical scope included countries in the Gulf region, Eastern Mediterranean region, and North Africa for which published data concerning *C. auris* were available. Studies were considered eligible when they reported original data involving *C. auris* from the predefined geographical area and provided information relevant to one or more of the following domains: epidemiology, reported cases, clinical presentation, colonization, infection, laboratory identification, antifungal susceptibility or resistance, molecular typing/clade distribution, outbreaks, transmission, or clinical outcomes. Eligible study designs included case reports, case series, outbreak investigations, retrospective and prospective observational studies, surveillance studies, epidemiological investigations, and molecular epidemiological studies. Review articles, systematic reviews, meta-analyses, and other secondary publications were not considered primary evidence; however, their reference lists were screened manually to identify additional relevant primary studies. Records retrieved from the databases were screened for relevance based on their titles and abstracts. Potentially eligible articles were subsequently assessed through full-text examination. Duplicate records identified across databases were removed before or during the screening process. Studies were excluded when they did not concern *C. auris*, did not provide data from the target geographical area, did not contain relevant epidemiological or clinical information, or did not provide original regional data.

## 3. *C. auris* in the Middle East

### 3.1. Gulf Region

#### 3.1.1. Saudi Arabia

**Figure 1:**
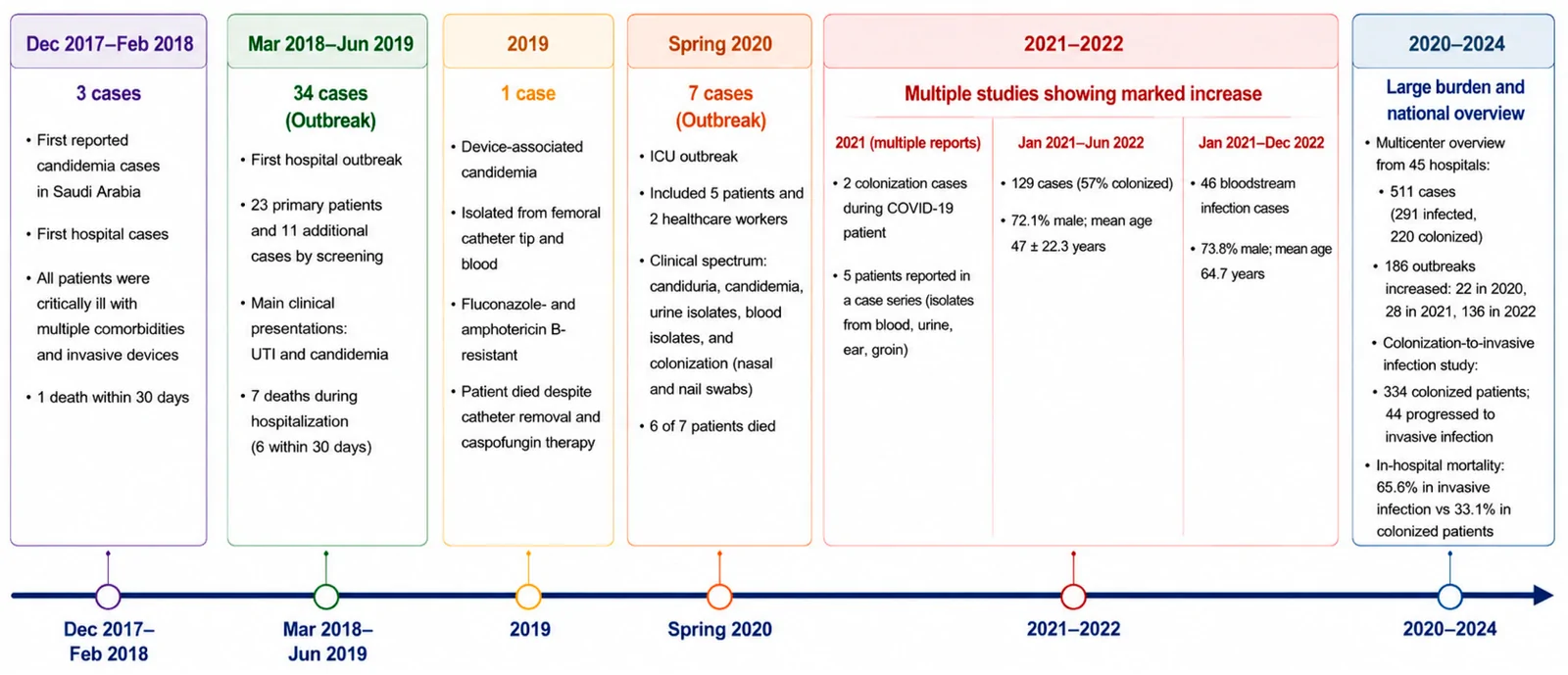
Timeline of *C. auris* cases in Saudi Arabia by date of occurrence. Arrows refer to the study date.

The first *C. auris* candidemia case in Saudi patients was reported between December 2017 and February 2018 (Figure 1) (Table 1) [16]. The isolates were obtained from bloodstream and pleural fluid. These early three Saudi cases were notable because *C. auris* was initially misidentified as *C. haemulonii* by the Vitek 2 system, illustrating the diagnostic limitations of conventional phenotypic identification. All isolates were fluconazole-resistant, and some also showed elevated amphotericin B MICs, while confirmation by MALDI-TOF MS and whole-genome sequencing established that they belonged to the South Asian clade. The patients were all critically ill women with multiple comorbidities, invasive devices, recent surgery, and prolonged hospitalization, which strongly supports the role of healthcare-associated acquisition. Although echinocandins or liposomal amphotericin B achieved microbiological clearance in most cases, one patient died within 30 days, reinforcing the high clinical severity of *C. auris* candidemia in Saudi intensive care settings [16].

The second Saudi case was reported in 2019 and involved a severely debilitated, bed-bound elderly diabetic patient with prolonged hospitalization, initial ICU admission, tracheostomy, femoral line placement, and multiple hospital-acquired infections [17]. *C. auris* was recovered from the femoral catheter tip and peripheral blood cultures, then confirmed by MALDI-TOF MS after initial detection by Vitek 2. The isolate was resistant to both fluconazole and amphotericin B, and the patient ultimately died despite catheter removal and initiation of caspofungin. This case further highlights the strong association of *C. auris* with device-related bloodstream infection, multidrug resistance, and poor clinical outcome in high-risk Saudi patients.

The first outbreak in a tertiary-care hospital in Saudi Arabia was reported during March 2018–June 2019 [18]. The study detected 23 primary patients and 11 additional cases identified by postexposure screening. Infections were more common than colonization, with urinary tract infection and candidemia as the main clinical syndromes. Seven patients died during hospitalization, and 6 died within 30 days of diagnosis.

Another study reported a single-center series of 7 patients with *C. auris* isolated from bloodstream and wound specimens during 2018–2019. Organism identification was performed by MALDI-TOF MS, and whole-genome SNP analysis was used for molecular typing. The genomic data showed that all isolates belonged to the South Asian clade, supporting hospital transmission within the center. Several isolates carried the *ERG11 K143R* substitution, a mutation associated with azole resistance. Antifungal susceptibility testing showed universal fluconazole resistance, with frequent amphotericin B non-susceptibility/resistance, whereas the isolates remained susceptible to voriconazole, posaconazole, and echinocandins. Three patients died out of seven patients. This study confirmed hospital transmission and Clade I presence [19]. During spring 2020, a 7-case outbreak of *C. auris* took place in the ICU of King Khalid Hospital, Saudi Arabia. Five patients plus two healthcare workers tested positive during the outbreak investigation. The outbreak included candiduria, candidemia, urine isolates, blood isolates, and colonization detected from nasal and nail swabs, indicating a mixed spectrum of colonization and infection. Identification and species confirmation were performed by the microbiology laboratory and the Vitek 2 YST card, with susceptibility testing also performed by the Vitek 2 YST card at the regional laboratory. The study reported high *C. auris* infection mortality, as six of the seven affected patients died [20].

In the following year (2021), a study reported 2 cases of *C. auris* in a university hospital in Saudi Arabia. Both patients were polymorbid, had prolonged hospital stays and recurrent ICU admissions, and *C. auris* was isolated from urine cultures, indicating colonization rather than invasive infection. Identification was performed by VITEK-2, with confirmation by MALDI-TOF MS. The isolates also grew well at 42 °C. One patient died after one month of *C. auris* isolation, while the other died before treatment was initiated [21]. In the same year, a study reported 1 case of *C. auris* candidemia in a 48-year-old woman admitted to the ICU with severe COVID-19 pneumonia and multiple comorbidities. The organism was identified by VITEK-2, and growth at 42 °C supported the identification. Anidulafungin was started after blood culture positivity on day 20 of admission, but the patient remained critically ill and died on day 90 from cardiorespiratory arrest [22]. Another study published in the same year described 6 *C. auris* isolates from 5 hospitalized patients in a tertiary-care hospital in Eastern Saudi Arabia. The specimens were obtained from blood, urine, ear swab, and groin screening samples. Identification was performed by culture at 42 °C followed by MALDI-TOF MS. The study also sequenced 18S rRNA and ITS regions for phylogenetic analysis, with additional *ERG1*1 and *FKS1* sequencing to assess resistance-associated mutations. The authors reported universal fluconazole resistance and detected *ERG11 F132Y* and *K143R* substitutions, whereas *FKS1* was mutation-free. The affected patients were reported to be critically ill and had died from severe comorbid illness [23].

An important study published in 2023 conducted a retrospective single-center analysis including 27 patients with invasive *C. auris* identified between 2015 and 2022 [24]. Clinical presentations were dominated by central line–associated bloodstream infections, followed by urinary tract infections, in addition to intra-abdominal and skin/soft tissue infections. Identification was performed using MALDI-TOF MS, and antifungal susceptibility testing utilized broth microdilution. The susceptibility profile demonstrated preserved activity of echinocandins, amphotericin B, and voriconazole, whereas fluconazole resistance was substantial. Clinical outcomes showed high mortality, as 18 patients died out of the 27 infected patients. Epidemiologically, the authors reported a progressive annual increase in invasive *C. auris* cases, although no data were available to distinguish colonization from true infection, limiting interpretation of disease burden. Notably, all invasive cases were confined to 2021–2022, with no cases detected during the pre-COVID-19 period (2015–2019). This temporal clustering suggests a post-pandemic emergence, potentially reflecting undetected outbreaks during the COVID-19 period, when infection control surveillance may have been compromised due to healthcare system strain.

A study published in 2023 described the occurrence of *C. auris* in a tertiary hospital in Riyadh using a retrospective dataset collected between November 2019 and December 2022. The study included 53 confirmed *C. auris*-positive cases, of whom 33 were male, and the age range was 15–98 years. Urine yielded the largest number of positives, and only one case met the definition of hospital-acquired infection/colonization; the remaining cases were detected on admission or through surveillance screening. The authors noted that all cases had at least one risk factor, with healthcare exposure being the strongest. Further, the authors also emphasized that *C. auris* may be under-recognized locally and that the first case in their institution appeared in November 2019, with additional cases accumulating during the COVID-19 era [25]. Another report was published in the same year and analyzed *C. auris* cases at King Saud Medical City, Riyadh, during January 2021–June 2022. The study identified 129 cases in total, and the cohort was predominantly male (72.1%). The mean age was 47 ± 22.3 years, and 57% were colonized and detected through active screening rather than clinical disease. The most frequent colonization sites were axilla/groin and nasal swabs, and the study also noted that 78.3% of patients were in critical care, 73.6% had vascular catheters, 88% had urinary catheters, and 66.7% required mechanical ventilation. The authors documented a multidisciplinary outbreak investigation and a bundle of mitigation measures that successfully contained transmission. They found that mortality was associated on univariate analysis with several ICU-related factors, while Foley catheter use remained the only independent predictor of mortality in multivariate analysis [26].

A study published in 2024 evaluated 46 patients with *C. auris*-positive blood cultures at King Abdulaziz Medical City, Jeddah, between January 2021 and December 2022. All cases were healthcare-associated, with a mean age of 64.67 years and 73.9% male predominance. The most frequent concurrent hospital-acquired infections were skin and soft tissue infection, hospital-acquired pneumonia, and intra-abdominal infection [27]. The study also showed that major predisposing factors were prolonged antibiotic exposure, prior *C. auris* colonization, central venous catheter use, ICU admission, mechanical ventilation, total parenteral nutrition, prior intra-abdominal surgery, and immunosuppressive therapy. Identification was performed by MALDI-TOF MS. The isolate profile was strongly resistant to azoles, as 97.8% were fluconazole-resistant, and 17.4% were amphotericin B-resistant. The paper reported one case each of endocarditis and endophthalmitis, and the 30-day all-cause mortality was 47.8%.

A recent important study conducted a retrospective case–control study in a tertiary-care hospital in Saudi Arabia evaluating 334 patients colonized with *C. auris* between 2018 and 2024. Among these, 44 patients progressed to invasive infection, while the remainder remained colonized [28]. The study focused on the transition from colonization to invasive disease, rather than describing only infected cases. Invasive infections were associated with central line use, mechanical ventilation, parenteral nutrition, and recent abdominal surgery, the latter being the only independent predictor of progression. Clinical outcomes demonstrated a marked difference between groups. In-hospital mortality was significantly higher in the infection group (65.9%) compared to colonized patients (33.1%), indicating that progression to invasive disease substantially increases lethality [28]. A retrospective multicentric overview of healthcare-associated *C. auris* outbreaks in 45 Ministry of Health hospitals in Saudi Arabia during 2020–2022 was published recently [29]. The dataset included 511 cases, of which 291 were classified as infected and 220 as colonized. Most cases were from the ICU; 68.9% were male, 32.9% were older than 65 years, and the common device-associated risk factors were Foley catheter use, mechanical ventilation, and central line insertion. The crude mortality rate was 41.5%, and mortality was numerically higher in infected than colonized patients. The authors reported 186 outbreaks in total, with a marked increase over time, as 22 outbreaks were in 2020, 28 outbreaks were in 2021, and 136 outbreaks were in 2022. Geographically, the central province had the highest burden, followed by the western and eastern provinces. Colonization was most often detected in axilla/groin/nasal swabs, while the most frequent clinical specimens were swabs and blood. The study emphasizes that early surveillance, strict infection prevention and control, and prospective follow-up after index cases were key containment measures [29]. Another recent study investigated a *C. auris* outbreak in a tertiary-care hospital in Riyadh, Saudi Arabia, analyzing 20 patients with 23 isolates collected between 2018 and 2019 [30]. The study focused on healthcare-associated transmission, with isolates derived from clinical and/or surveillance samples. Identification and characterization were performed using WGS, followed by SNP-based phylogenetic analysis. The data demonstrated that isolates clustered into 4 different clades, indicating multiple introductions into the hospital rather than a single-source outbreak. The genomic distances between isolates supported nosocomial transmission within clusters. Further, the study reported mutations in the resistance-associated gene *ERG11*, consistent with azole resistance. Interestingly, the key contribution of this study is the demonstration that *C. auris* epidemiology in Saudi Arabia is not attributable to a single clonal expansion, but rather to multiple introductions with subsequent intra-hospital spread, emphasizing the importance of genomic surveillance in outbreak settings. Another crucial point revealed by the study is that the retrospective application of molecular tools, specifically PCR and WGS, was essential for accurately delineating the true extent of the outbreak, revealing cases that were previously unrecognized. Although updates to MALDI-TOF MS and automated system libraries have improved current diagnostic accuracy, the findings underscore the necessity for ongoing molecular surveillance and systematic re-analysis of archived isolates in outbreak investigations [30].

Chronological summary of reported *C. auris* events in Saudi Arabia from December 2017 to 2024. The timeline presents the date or period of case occurrence and the number of cases.

**Table 1:** First documented reports of *C. auris* in Middle Eastern countries.

Country	Year of First Report	Type of Report	Cases	Clade	Reference
Kuwait	2014	Candidemia case	1	I	[31]
Israel	2014	Clinical case	4	III-related lineage later predominated	[32]
Oman	2016	Candidemia cases	2	I	[33]
Saudi Arabia	2018	Candidemia	3	I	[16]
UAE	2018	Persistent candidemia	1	Not reported	[34]
Qatar	2018	Colonization/outbreak index case	1	I	[35]
Sudan	2019	Blood samples collected from febrile patients	26	Not reported	[36]
Lebanon	2020	ICU outbreak	14	I	[14]
Jordan	2021	Single case	1	Not reported	[37]
Bahrain	2021	Hospital outbreak	59	Not reported	[38]
Egypt	2021–2022	Clinical isolates	8	Not reported	[39]

#### 3.1.2. Qatar

The first *C. auris* outbreak in a tertiary-care general hospital in Qatar was reported in 2018 [35]. It involved 13 patients with either infection or colonization. The index case was first detected in November 2018 in a patient colonized in the respiratory tract. Subsequent cases occurred in the medical ICU and a medical inpatient unit, with epidemiologic linkage suggesting ward-based transmission, which later expanded the outbreak investigation. The outbreak was ultimately controlled through a bundled infection prevention and control response that included patient screening and decolonization, environmental screening, enhanced disinfection with peracetic acid wipes, 1% chlorine, and hydrogen peroxide vapor, contact precautions, and enhanced hand hygiene. The outbreak was declared terminated on 22 August 2019 [35]. Another study covered the same period. The study included 13 patients with confirmed *C. auris* infection/colonization: 5 true infections and 8 colonized patients. The infected group was older, with a mean age of 76.6 years, than the colonized group, with a mean age of 66.4 years. Among the infected patients, the clinical syndromes were 2 urinary tract infections, 1 candidemia, 1 soft tissue infection, and 1 lower respiratory tract infection. It was noted that all patients had prior or concurrent bacterial/viral illness, indicating a heavily comorbid, critically ill population [40]. Identification was performed by Vitek 2 XL. Antifungal susceptibility testing showed that all the isolates were susceptible to echinocandins, flucytosine, and posaconazole, but resistant to fluconazole and amphotericin B. For outbreak investigation, the authors used PFGE/electrophoretic karyotyping, which supported transmission within the critical care and medical units; screening identified the 8 additional colonized patients. Among the 5 infected patients, 3 died during antifungal therapy [40]. Later, Salah et al. performed a genomic epidemiological investigation of *C. auris* in Qatar using 44 isolates/samples collected from three tertiary-care hospitals and the hospital environment between 1 April 2018 and 30 November 2020 [41]. These included 40 human isolates and 4 environmental isolates, derived from 36 patients; among them, 3 isolates were from invasive infections, and 37 were from colonized patients. Identification was performed on culture-grown isolates using MALDI-TOF MS. Whole-genome sequencing showed that all isolates belonged to the South Asian lineage. Most isolates clustered into a single major clade with very low genetic diversity, supporting ongoing nosocomial transmission. Four environmental isolates were genetically identical to corresponding clinical isolates, strengthening the inference that the hospital environment contributed to persistence and spread. The study also identified four genetically divergent clinical isolates, including the first reported Qatar case that had a travel history to Oman, and other divergent isolates from patients with healthcare exposure outside the local hospital network, suggesting multiple introductions into Qatar rather than a single-source event. Regarding resistance, the study reported *ERG11 Y132F* and *K143R* substitutions, both associated with fluconazole resistance. The main indication of the study is that *C. auris* in Qatar was already established as a South Asian lineage outbreak with hospital transmission and environmental persistence, and that genomic data were essential for distinguishing the predominant outbreak clone from additional imported or divergent strains [41]. In 2023, an important study performed a retrospective molecular epidemiology study of *C. auris* outbreak isolates in Qatar during the COVID-19 period. The analysis included 76 clinical isolates, of which 65 were from patients with COVID-19, collected between March 2020 and June 2021 from nine major hospitals. The study compared these isolates with earlier Qatari isolates reported from 2018 to 2020 [42]. Identification of isolates in the clinical workflow was performed by MALDI-TOF MS, and the sampled materials included 42 screening specimens, 14 blood isolates, 13 urine isolates, 4 respiratory isolates, and 3 wound isolates. Whole-genome sequencing showed that the outbreak was highly clonal, with low genetic variability among isolates from patients with and without COVID-19, supporting ongoing nosocomial dissemination across healthcare facilities. All Qatari isolates belonged to the South Asian clade. The study also reports that the Qatari outbreak isolates clustered with earlier local isolates, indicating persistence and spread within the healthcare system rather than repeated unrelated introductions. From a resistance perspective, the study found that all isolates were fluconazole-resistant except two with reduced MICs, and most of the isolates were amphotericin B-resistant. Echinocandin resistance emerged in a subset of isolates, with *FKS1* hotspot substitutions detected in three isolates. The study identified established resistance-associated substitutions, including *Y132F* in *ERG11* and *V704L* in *CDR1*, and described a particularly concerning isolate, CAS109, which was resistant to three antifungal classes and carried a premature stop codon in *ERG3* plus novel *CDR2* mutations [42]. Another study published in 2023 reported a retrospective study of *C. auris* bloodstream infection in the Hamad Medical Corporation network, Qatar, covering 1 November 2018 to 31 July 2021. The study identified 36 patients with *C. auris* fungemia by applying MALDI-TOF. Most were male, with a median age of 52 years, and nearly all were in the ICU, most after severe COVID-19 pneumonia [43]. Clinically, the infections were mostly central line-related; in 30 cases, a central line was considered the likely source, and 24 of those lines were promptly removed. Other sources included urinary catheter, pelvic collection, and secondary peritonitis. Three patients had recurrence of candidemia within 30 days. Antifungal susceptibility showed >90% fluconazole non-susceptibility and 85% amphotericin B resistance, whereas echinocandins remained nearly fully active. All patients received an echinocandin initially, and selected cases required voriconazole and/or flucytosine addition. The 30-day mortality was 41.6% [43]. A recent study conducted a retrospective multicenter observational study evaluating the epidemiology and clinical outcomes of *C. auris* across three tertiary-care hospitals in Qatar between May 2019 and December 2022. The study included 331 individuals with at least one positive *C. auris* culture [44]. The cohort was predominantly male with a median age of 56 years, and a large proportion had significant healthcare exposure and comorbidities. Patients were stratified into colonization versus invasive *C. auris* disease according to predefined criteria used by the original study. Among patients classified as having invasive disease, the most frequent sites were bloodstream (28.9%), urine (28.9%), and respiratory specimens (13.3%). Thus, the invasive-disease category in this study included infections identified from both bloodstream and non-bloodstream sites. The 30-day mortality was 37.8% among patients classified as having invasive disease, compared with 14.0% among colonized individuals. These mortality estimates reflect the classifications and outcome definitions used in the original study and should not be interpreted as fungemia-specific mortality. Clinically, ICAD cases were associated with greater healthcare intensity, including ICU admission, mechanical ventilation, central venous catheterization, and prolonged hospitalization. In multivariable analysis, mechanical ventilation emerged as the only independent predictor of invasive disease. Outcome analysis demonstrated a significant mortality burden. The 30-day mortality was 37.8% in ICAD patients, compared with 14.0% in colonized individuals, confirming that progression from colonization to invasive infection markedly increases lethality. Temporal analysis revealed peaks in reported cases during mid-2020 and mid-2021, coinciding with the COVID-19 period, suggesting pandemic-associated amplification of transmission and case detection [44].

#### 3.1.3. United Arab Emirates

The first report of *C. auris* from the United Arab Emirates (UAE) was published in 2018 as a case of persistent candidemia despite antifungal therapy [34]. The isolate was recovered from an elderly patient with multiple chronic comorbidities and prolonged ICU exposure, and it was initially misidentified by a conventional automated system as Candida haemulonii, leading to delayed recognition. Antifungal testing showed increasing MICs to several agents, and the organism persisted in the bloodstream until the patient died. This report was important because it documented the first UAE case and highlighted both the multidrug-resistant phenotype of *C. auris* and the diagnostic limitations of routine phenotypic systems in detecting this emerging pathogen [34]. In 2023, a retrospective national surveillance study examined the emergence of *C. auris* in the UAE by analyzing microbiology data collected from 2018 to 2021 across multiple reporting centers [45]. The study identified 908 non-duplicate *C. auris* isolates, with a marked annual increase from 9 cases in 2018 to 614 in 2021, indicating rapid expansion after the first detection period. Most isolates came from urine, blood, and skin and soft tissue specimens, and the burden was concentrated in hospital settings: 89% of patients were in inpatient care, and 45.6% were in intensive care units. Patients were predominantly adults, with a male preponderance. Clinical outcome analysis showed substantial mortality. Among cases with known outcomes, mortality was 27.5% for *C. auris* overall and 36.1% for blood-culture isolates. The study also estimated 17,706 excess hospital days attributable to *C. auris* over the full surveillance period, underscoring the healthcare burden. Antifungal resistance was prominent and increased over time. Fluconazole resistance reached 72.6% in 2021, amphotericin B resistance reached 84.6% in 2021, and resistance to caspofungin and micafungin emerged during the surveillance period. Among tested isolates, 47.67% were multidrug resistant, 3.89% were extensively drug resistant, and 1.17% were pandrug resistant. It can be concluded that *C. auris* circulation in the UAE expanded rapidly across all seven emirates and calls for strengthened infection control and surveillance [45]. Another single-center retrospective study from the UAE evaluated all Candida infections during 2022 and found 75 confirmed cases, of which 53 were *C. auris* [46]. The hospital prevalence of *C. auris* was 0.58% among 9125 admissions, and 92.5% of cases were hospital-acquired. Urine was the most common specimen source, while only 10 cases represented candidemia. Most patients were older adults with substantial comorbidity and ICU exposure. Mortality was high but not statistically different from non-auris candidemia [46]. A recent retrospective study assessed the value of universal *C. auris* screening on admission to an intensive care unit in the UAE [47]. It included 3356 ICU patients across two periods: a pre-screening phase involved 1658 patients, in the period between June 2020 and November 2021 and a post-screening phase that included 1698 patients, in the period between June 2022 and November 2023. Screening was performed using axilla and groin swabs, with identification by MALDI-TOF MS or VITEK 2 YST. Among screened patients, only 4 of 1698 were positive. The positivity rate did not differ significantly before versus after screening implementation, and interrupted time-series analysis showed no immediate or sustained effect of the program. Subgroup analysis in the medical ICU was similar. It can be concluded that universal admission screening detected few cases and suggested that targeted screening, together with periodic point-prevalence surveys and other infection-control measures, may be more efficient in this setting [47].

#### 3.1.4. Kuwait

Emara et al. described the first documented *C. auris* candidemia from Kuwait [31]. It is considered the earliest Gulf-region report, establishing the organism’s presence in the Arabian Gulf before later reports from Oman, Saudi Arabia, and UAE. The patient was a 27-year-old woman with chronic renal failure who developed bloodstream infection during severe illness and prolonged hospital care, illustrating the relevance of major host comorbidity and healthcare exposure. The yeast was initially recognized as *Candida haemulonii* by conventional laboratory methods, underscoring the risk of misidentification with routine phenotypic systems. Antifungal testing showed high-level fluconazole resistance, with a reported MIC of >256 μg/mL, while amphotericin B activity was relatively lower, and the patient died from multiorgan failure [31]. Another early report was published in 2018 that analyzed yeast isolates collected over a 3.5-year period from 2014 to 2017 [48]. The study confirmed 158 *C. auris* isolates from 56 patients using species-specific PCR and rDNA sequencing after many isolates were initially misidentified by Vitek 2 as *Candida haemulonii*. The isolates originated from bloodstream, urine, respiratory, and other clinical specimens, indicating broad healthcare-associated dissemination. Antifungal susceptibility testing showed universal fluconazole resistance, while 73% and 23% of isolates were additionally resistant to voriconazole and amphotericin B, respectively. Eleven isolates were multidrug-resistant to fluconazole, voriconazole, and amphotericin B simultaneously, and one isolate exhibited echinocandin resistance. The study highlighted the rapid emergence of *C. auris* as an important multidrug-resistant nosocomial pathogen in Kuwait and emphasized the limitations of conventional phenotypic identification systems [48]. Another report covered the same period [49]. A total of 314 *C. auris* isolates recovered from 126 patients between 2014 and 2018 were analyzed to investigate epidemiology, resistance, and transmission dynamics. The isolates were obtained from blood (58 isolates from 43 patients) and non-blood specimens (256 isolates from 83 patients), including urine, respiratory, wound, and screening samples. Importantly, all 212 isolates identified before 11 April 2018 were initially misidentified as *Candida haemulonii* until the Vitek 2 Compact database was updated, underscoring major diagnostic limitations in routine laboratories. Molecular typing demonstrated predominance of the South Asian clade (Clade I), supporting endemic healthcare-associated transmission. Antifungal susceptibility testing revealed universal fluconazole resistance with frequent amphotericin B non-susceptibility, whereas echinocandins largely retained activity. Resistance-associated *ERG11* mutations, particularly *Y132F* and *K143R*, were detected. Several isolates also exhibited multidrug-resistant phenotypes. Clinically, most affected patients had prolonged hospitalization, ICU exposure, invasive devices, and severe comorbidities, while invasive bloodstream infection was associated with considerable mortality [49]. In a molecular characterization study conducted at a tertiary-care hospital in Kuwait and published in 2022, 49 *C. auris* isolates obtained from 18 immunocompromised patients between June 2016 and December 2018 were analyzed to investigate echinocandin resistance mechanisms and molecular epidemiology [50]. The isolates were recovered from bloodstream, urine, respiratory, and other clinical specimens, representing candidemia, candiduria, and invasive healthcare-associated infections. Short tandem repeat typing demonstrated that all isolates belonged to the South Asian clade (Clade I), supporting nosocomial transmission within the institution. Antifungal susceptibility testing revealed that most isolates were highly resistant to fluconazole, while some also exhibited amphotericin B resistance. Importantly, 12 isolates from 8 patients developed reduced echinocandin susceptibility, and sequencing identified multiple *FKS1* hotspot mutations, including a novel *R1354H* mutation associated with echinocandin resistance emerging during antifungal therapy. The study highlighted therapy-driven evolution of multidrug resistance in severely immunocompromised hosts. Clinically, affected patients had major comorbidities and prolonged hospitalization, and 10 of the 18 patients died, indicating substantial mortality associated with invasive *C. auris* infection [50]. Finally, a case report published in 2022 investigated an immunocompromised patient with a lung transplant who developed breakthrough *C. auris* candidemia. Seven isolates were obtained from urine, respiratory, and blood over 23 days. All were fluconazole-resistant with the *ERG11 K143R* mutation. They were susceptible to amphotericin B, while Echinocandin MICs diverged. The early colonizing isolates had low micafungin/anidulafungin MICs, whereas the bloodstream and late respiratory isolates showed raised micafungin MICs with novel *FKS1* hotspot-1 mutations (S639T or S639Y) and the patient died eventually [51]. Another earlier article described an 18-month *C. auris* outbreak in the high-dependency unit of a secondary-care hospital in Kuwait, with the outbreak likely beginning in January 2018 and being recognized in September 2018 after three consecutive candidemia cases [52]. The study included 71 affected patients in total, 17 with candidemia and 54 colonized patients. Molecular typing showed that the outbreak isolates were genetically indistinguishable and belonged to the South Asian clade I, supporting sustained nosocomial transmission rather than repeated unrelated introductions. Antifungal resistance was extensive: nearly all isolates were fluconazole-resistant, and only urine isolates from two patients developed echinocandin resistance with concomitant FKS1 mutations. Despite treatment of all candidemia cases and most colonized patients, 9 of 17 candidemia patients and 27 of 54 colonized patients died, giving an overall crude mortality of about 50%. The study highlighted both environmental persistence and the need for strict infection-control measures [52].

#### 3.1.5. Oman

Reports of *C. auris* in Oman describe a growing number of healthcare-associated cases and outbreaks with severe drug resistance and high mortality. The earliest report documented two candidemia cases but offered limited data beyond noting that isolates initially misidentified as *C. haemulonii* had very high MICs to fluconazole and amphotericin B [33]. Subsequent investigations confirmed that all isolates belonged to the South Asia lineage. For example, Al Maani et al. (2019) [53]. analyzed a large outbreak in Sohar between April 2018 and April 2019, involving 32 patients with a median age of 64 years, including 11 with candidemia, 14 with symptomatic urinary infections, and 7 asymptomatically colonized. All clinical and environmental isolates in this outbreak were typed as clade I by ITS sequencing, and nearly all were multidrug-resistant, as 100% had elevated fluconazole MICs, 33% had amphotericin B MICs in the resistant range, whereas MICs for echinocandins and voriconazole remained uniformly low. The crude 30-day mortality in this Sohar series was 53.1% [53]. In parallel, another study reported 23 *C. auris* candidemia cases at the Royal Hospital (Muscat) from 2016–2018. Patients were mostly older (21 adults, 2 pediatric) with typical ICU risk factors. All isolates again belonged to clade I by short tandem repeat genotyping. In vitro, every isolate was non-susceptible to fluconazole, and many had high amphotericin B MICs, but all had low echinocandin MICs. First-line therapy with caspofungin or anidulafungin was uniformly used. The crude mortality in this Muscat series was 39.1%. Notably, none of these reports identified specific resistance mutations in *ERG11* or *FKS1*, but the clade I assignment is consistent with global data on resistant *C. auris* [54]. Most recently, Al Mamari et al. (2026) [55]. reviewed all *C. auris* isolates at a tertiary referral hospital between 2017 and 2021. A total of 129 patients were identified, of whom 51 had invasive infection, and 78 were colonized. Among infected patients, candidemia accounted for 76.5% and was associated with 49% mortality. Susceptibility testing in this cohort showed 100% fluconazole resistance and very high amphotericin B resistance, and only 5.2% of isolates were susceptible, whereas voriconazole susceptibility was intermediate (44.8%). By contrast, echinocandins retained near-universal activity, and flucytosine was largely active, as 80% of the cases were susceptible [55]. These findings reinforce a profile of pan-azole/non-echinocandin resistance in Oman *C. auris*.

#### 3.1.6. Bahrain

In Bahrain, the first large-scale reports of *C. auris* describe a hospital outbreak that began in late 2021. Alagha et al. (2024) [38]. reported that 59 patients were seen at the Salmaniya Medical Complex during the 2021–2022 outbreak. The data showed that the mean age was 63.9 years and 74.6% of the patients were male. These patients were predominantly critically ill with typical risk factors such as central lines, antibiotics, and intubation, and the crude mortality was 44.1% [38].

In a subsequent three-year candidemia survey from 2021 to 2023, Saeed et al. (2024) [56] identified 430 candidemia cases. The mean age was 65.7 years, with a mortality rate of 85.5%. *C. auris* accounted for 78 of 430 isolates (18%). In that series, all *C. auris* isolates remained susceptible to amphotericin B, but were essentially pan-azole-resistant. Echinocandin activity was retained in nearly all isolates [56]. Sowar et al. (2026) similarly analyzed 323 patients from 2021 to 2025 and found that every isolate was non-susceptible to fluconazole and many had elevated amphotericin B MICs (MIC50 = 2, MIC90 = 4 mg/L), whereas echinocandin MICs remained in the wild-type range in all cases [57]. None of the Bahrain studies reported genotyping or clade assignment; based on regional analyses, Bahraini isolates are presumed to belong to clade I as seen in Oman and neighboring Gulf countries. Overall, the literature shows that Bahraini *C. auris* cases have involved mostly elderly or critically ill patients in tertiary hospitals, with very high crude mortality and a multidrug-resistant profile.

### 3.2. The Eastern Mediterranean Region

*C. auris* has emerged in the Eastern Mediterranean region over the past decade. In Lebanon, the first known cases were a 2020–2021 hospital outbreak at the American University of Beirut Medical Center (AUBMC). Allaw et al. (2021) [14] reported 14 ICU patients identified over 13 weeks beginning in October 2020. All patients had serious comorbidities and prior broad-spectrum antibiotic exposure; half had concurrent COVID-19. *C. auris* was recovered from blood, urine, respiratory samples and skin. Identification was performed by MALDI-TOF and Vitek-2. Six patients received caspofungin or amphotericin B, while eight were deemed colonized [14]. A subsequent case-control study at the same center (October 2020–June 2021) identified 56 ICU patients with *C. auris*, of whom 32 had severe COVID and 24 without. High quick-SOFA score, prolonged ICU stay, and presence of central or urinary catheters were independent risk factors for *C. auris* acquisition among COVID-19 patients. Thirty-day mortality was linked to high *Candida* score and septic shock; antifungal therapy ≥7 days improved survival [58]. Parallel molecular analysis of 28 isolates from 21 patients collected in various ICU units confirmed that all belonged to South Asian clade I. This work used MALDI-TOF, Vitek 2, confirmatory ITS/D1–D2 PCR sequencing, and long-read WGS. All isolates were uniformly resistant to fluconazole and amphotericin B, yet fully susceptible to echinocandins. Notably, *ERG11* had the *Y132F* mutation and the efflux pump gene *CDR1* carried a novel *D709E* substitution. Phylogenomics revealed minimal diversity among Lebanese isolates, consistent with clonal person-to-person transmission of clade I [59]. In Israel, *C. auris* was first detected in 4 patients during 2014 [32]. In 2017, a single-hospital outbreak was traced to a patient repatriated from South Africa [60]. In recent years, Israel has experienced a massive surge: a nationwide survey (2014–2022) identified 209 patients, both clinical and colonized [13]. The reported cases jumped 30-fold in 2021, coinciding with COVID-19 hospitalization waves. Multilocus sequence typing showed multiple, hospital-level clusters. A South African clade III clone that was likely introduced in 2016 became predominant after 2021, accounting for 49% of sequenced isolates. This clade III strain exhibited 100% fluconazole and 74% voriconazole resistance, far higher than non-clade-III isolates. Twenty-three percent of Israeli *C. auris* patients had COVID-19 and 78% required mechanical ventilation. Outbreaks initially involved ventilated COVID-19 ICU patients, then spread to ventilated non-COVID patients, and then to other wards. Thus, the Israeli pattern reflects global reports of *C. auris* flourishing amid COVID-19 ICU conditions.

Finally, in Jordan, the first and only reported case to date occurred in June 2021. The study describes a 48-year-old diabetic woman who was recently hospitalized for COVID-19, developed sepsis and had *C. auris* in urine on day 4 [37]. Broth microdilution in 2023 showed the isolate was susceptible to all tested antifungals. The patient died of sepsis complicated by carbapenem-resistant *K. pneumoniae* before antifungal susceptibility was even reported.

### 3.3. North Africa

#### 3.3.1. Egypt

Published Egyptian studies on *C. auris* are few and recent. Early surveillance found no *C. auris* among hundreds of *Candida* isolates, suggesting rarity until the mid-2020s. In more detail, Khairat et al. (2021) [61] performed a two-year screening of 400 Candida isolates from Cairo University Hospital. They used chromogenic media, growth at 42 °C, and MALDI-TOF for species ID. Most isolates were *C. albicans*, and no *C. auris* were found [61]. Mahmoud et al. (2024) [39] conducted a prospective cross-sectional study of 223 Candida isolates collected from April 2021 to October 2022 at Cairo University Hospitals. They recovered *Candida* from urine, blood, catheter tips, and sputum. Identification by germ-tube test, chromogenic agar, and Vitek-2 was supplemented by MALDI-TOF for thermotolerant isolates. They found *C. auris* eight isolates: five from blood cultures, two from central venous catheters, and one from urine. Antifungal susceptibility showed that all eight isolates were susceptible to voriconazole but non-susceptible to caspofungin and amphotericin B. The molecular methods included Vitek-2 identification and ITS sequencing for confirmation [39]. Another study investigated the distribution and antifungal susceptibility profiles of *Candida* species causing candidemia among 90 patients admitted to Cairo University tertiary hospitals between October 2021 and January 2023. Species identification was performed using MALDI-TOF MS, followed by antifungal susceptibility testing using the VITEK 2 Compact system. Among 44 adult candidemia cases, *C. auris* accounted for 3 isolates (6.8%), whereas no *C. auris* isolates were detected among the 46 pediatric cases. Importantly, all three *C. auris* isolates demonstrated resistance to every antifungal agent tested, including fluconazole, voriconazole, caspofungin, micafungin, amphotericin B, and flucytosine, making *C. auris* the most resistant species identified in the study [62]. Two 2025 publications expanded on Egyptian *C. auris*. The first is a study performed in an 18-month epidemiological study at a Cairo tertiary hospital. They started with 140 antifungal-resistant *Candida* isolates and identified 120 of them as *C. auris*. The patients were predominantly male and older than 60 years. The clinical sources were mainly sterile-site infections: 60.8% bloodstream, 20.8% respiratory, 15% urinary, and 3.4% other fluids. Common risk factors were liver transplant, recent COVID-19, and malignancy. Crucially, antifungal susceptibility for these 120 isolates showed universal fluconazole resistance. In contrast, most isolates remained sensitive to amphotericin B and echinocandins. Only 4 out of 120 were caspofungin-resistant, and 5 were micafungin-resistant. Voriconazole and posaconazole showed uniformly low MICs in this collection [63]. The other study reported molecular characterization of four multidrug-resistant *C. auris* from Egypt. They selected test strains from previously identified clinical isolates. Identification was confirmed by ITS sequencing and phylogenetic analysis. Antifungal susceptibility was assessed for each. Molecular sequencing of *ERG11*, *ERG3*, *FKS1*, and *FKS2* revealed *ERG11 Y132F* in all strains, conferring azole resistance and FKS2 F635Y in some, conferring echinocandin resistance. These mutations are well-known in clade I/III *C. auris* [64]. All studies performed in Egypt did not mention clade type.

#### 3.3.2. Algeria

Zerrouki et al. reported the first documented series of *C. auris* in Algeria, describing seven cases of infection or colonization detected in intensive care units, which establishes the organism as an emerging nosocomial threat in North Africa. The isolates were recovered from bronchial aspirates, wound swabs, urine, and peritoneal fluid, and species confirmation was achieved by MALDI-TOF MS together with ITS and D1/D2 sequencing. Antifungal susceptibility testing showed reduced activity of several azoles, supporting the multidrug-resistant phenotype that characterizes *C. auris* and complicates empirical therapy in critically ill patients [65].

#### 3.3.3. Sudan

In Sudan, a study reported *C. auris*-positive results in 26 of 100 febrile patients based on blood PCR. However, this unusually high positivity rate should be interpreted cautiously, as the finding was based on a single study and molecular detection alone does not necessarily establish confirmed *C. auris* infection or represent population-level prevalence. Accordingly, this report is considered preliminary evidence of detection rather than a reliable estimate of *C. auris* prevalence in Sudan [36].

## 4. Discussion

### 4.1. Comparative Reported Cases Across North Africa, the Gulf Region, and the Eastern Mediterranean Region

**Figure 2:**
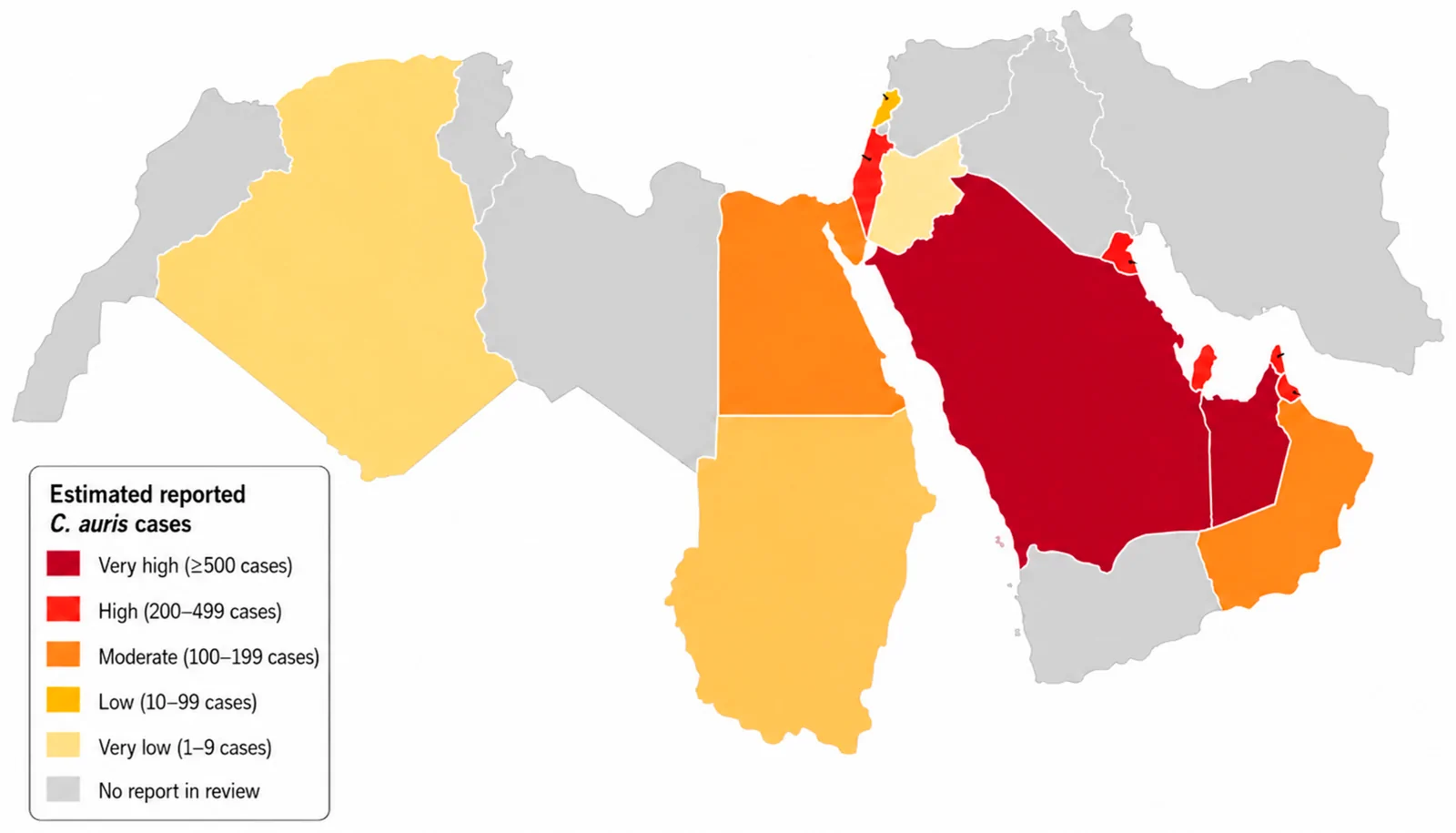
Geographic distribution of reported *C. auris* cases in the Middle East.

The reported cases of *C. auris* show marked regional heterogeneity across North Africa, the Gulf region, and the Eastern Mediterranean region (Figure 2). The Gulf region currently carries the highest documented burden, with repeated hospital outbreaks and large case series reported from Saudi Arabia, Qatar, UAE, Kuwait, Oman, and Bahrain (Table 2). In Saudi Arabia, early cases were first described in 2018, followed by multiple outbreak investigations and larger surveillance studies, including 511 infected or colonized patients and 186 outbreaks between 2020 and 2022 [29]. Qatar similarly reported sustained hospital transmission, including an initial outbreak of 13 patients and later multicenter studies showing 331 individuals with positive cultures and clearly reported case peaks during the COVID-19 period [35,40,42,44]. The UAE showed rapid expansion, with 908 non-duplicate isolates identified between 2018 and 2021 [45,46]. Kuwait and Oman likewise demonstrated substantial healthcare-associated circulation, with outbreak cohorts and candidemia series showing ongoing transmission and high mortality [48,52,55]. In the Eastern Mediterranean region, the burden is substantial but more heterogeneous. Lebanon reported its first major outbreak during the COVID-19 era, involving 14 ICU patients, followed by larger epidemiologic and genomic studies confirming ongoing hospital transmission [14,58,59]. Israel experienced the most dramatic regional increase, with a nationwide outbreak driven by COVID-19 hospitalizations and a 30-fold rise in 2021 [13]. Jordan, by contrast, has reported only a single case to date [37]. Thus, the Eastern Mediterranean region demonstrates clear post-pandemic amplification of *C. auris* but generally with fewer published cases than the Gulf region, except for Israel.

North Africa appears to have the lowest reported burden, although the available evidence suggests recent emergence rather than true absence. In Egypt, early surveillance found no *C. auris* among 400 Candida isolates, whereas later studies detected 8 isolates in one cohort, 3 isolates among adult candidemia cases, and then 120 isolates in a single tertiary-hospital [62]. Algeria has also reported a small ICU series of seven cases [65]. Compared with the Gulf and Eastern Mediterranean regions, North Africa therefore shows a later and more limited published burden, although under-detection likely contributes to the apparent low reported cases.

**Figure 3:**
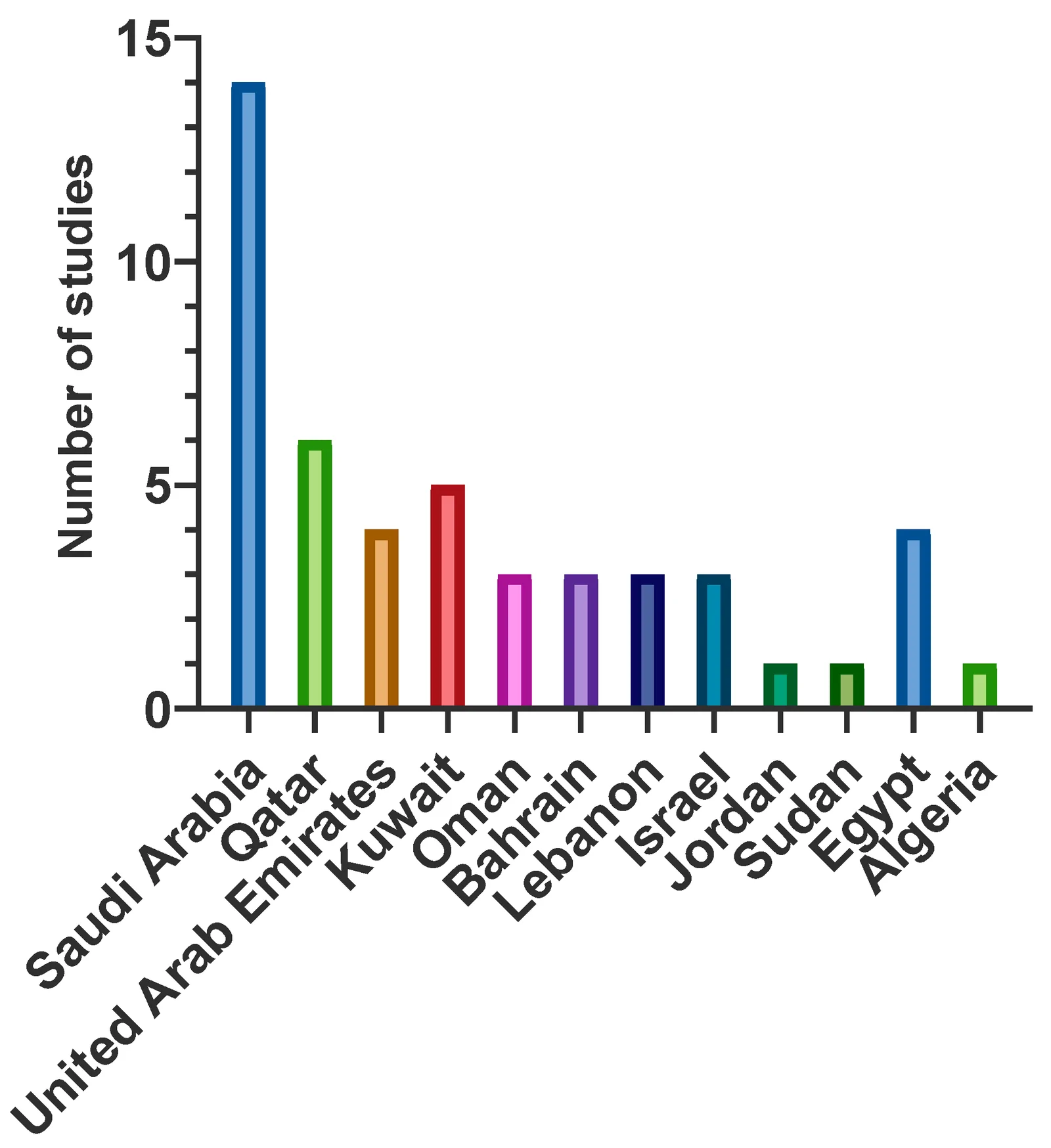
Distribution of published *C. auris* studies across Middle Eastern countries.

The observed regional variation in *C. auris* reported cases is likely multifactorial rather than purely biological. In the Gulf region, the high burden probably reflects a combination of earlier recognition, stronger diagnostic capacity, intensive hospital surveillance, and a large pool of critically ill patients with prolonged ICU exposure, invasive devices, and repeated healthcare contact. In addition, Saudi Arabia deserves particular emphasis because the literature currently contains the largest number of published *C. auris* studies in the Middle East, with 14 Saudi reports summarized in the Saudi subsection (Figure 3). This concentration of publications most likely reflects both a substantial epidemiologic burden and comparatively strong surveillance, diagnostic capacity, and outbreak recognition. In Saudi Arabia, early cases were first described in 2018, followed by multiple outbreak investigations and larger surveillance studies, including 511 infected or colonized patients and 186 outbreaks between 2020 and 2022. This pattern indicates not only repeated introductions and sustained nosocomial transmission, but also a level of documentation that exceeds that of neighboring countries. Saudi Arabia has an important epidemiologic context not shared to the same extent by any Middle Eastern countries. The Hajj and Umrah mass gatherings bring very large numbers of pilgrims from many countries into dense congregate settings, and WHO classifies such mass gatherings as events with substantial infectious-disease transmission potential. This illustrates the scale of repeated population mixing that can facilitate importation, amplification, and geographic spread of healthcare-associated pathogens once they enter hospitals. This does not prove a direct causal link for *C. auris*, but it is a plausible contributor to the breadth of Saudi case detection and the diversity of outbreak settings.

By contrast, the lower number of reported North African cases most likely reflects a combination of later emergence and under-detection. The diagnostic delays, under-resourced laboratories, and limited surveillance can allow colonized patients to remain unrecognized and continue transmission. Therefore, the smaller North African case count should not be interpreted as absence of transmission; rather, it likely reflects both genuine epidemiologic delay and weaker detection infrastructure compared with the Gulf region. In the Eastern Mediterranean region, the intermediate burden appears to be driven by similar healthcare-associated transmission dynamics, but with greater heterogeneity between countries and strong acceleration during the COVID-19 period.

Geographic distribution of reported *C. auris* cases in the Middle East based on data extracted from the studies included in this review. Countries were categorized according to the estimated number of reported cases. Reported case numbers are approximate and should not be interpreted as national reported case estimates because data were derived from heterogeneous studies, outbreaks, and surveillance reports.

Number of published studies reporting *C. auris* cases in countries of the Middle East. Saudi Arabia contributed the highest number of publications, followed by Qatar and Kuwait, whereas fewer studies were identified from the rest of the countries. The figure highlights the uneven geographical distribution of *C. auris* research and reporting across the region, reflecting differences in surveillance capacity, diagnostic infrastructure, publication output, and outbreak recognition. Data were extracted from studies included in the present review.

### 4.2. Pre-COVID vs. Post-COVID Emergence

In the Middle East, *C. auris* was largely absent before 2020, with emergence clustered in the COVID era. In Saudi Arabia, the earliest reports consisted of isolated cases and relatively small outbreaks. The first recognized infections were reported between late 2017 and early 2018, followed by a tertiary-care outbreak involving 23 primary patients and 11 additional screened cases during 2018–2019 [16,18]. Another single-center study identified only seven patients during 2018–2019 [19]. However, subsequent investigations demonstrated a substantial increase in disease burden after the onset of the pandemic. A retrospective analysis covering the period 2015–2022 identified 27 patients with invasive *C. auris* infection and notably reported that all invasive cases occurred during 2021–2022, with no invasive cases detected during 2015–2019 [24]. At the national level, a multicentric analysis documented 511 infected or colonized patients and 186 outbreaks between 2020 and 2022, with outbreak numbers increasing dramatically from 22 in 2020 to 136 in 2022, illustrating an unprecedented expansion during the pandemic and immediate post-pandemic years [29].

A similar temporal pattern was observed in Qatar. The first recognized outbreak involved only 13 patients and was linked to transmission occurring between 2018 and 2019 before publication in 2020 [35,40]. Subsequent molecular epidemiological investigations during the COVID-19 period demonstrated extensive dissemination across healthcare facilities. Among 76 outbreak isolates collected between March 2020 and June 2021, 65 were recovered from patients with COVID-19, indicating a strong temporal overlap between severe COVID-19 hospitalizations and *C. auris* transmission [42]. Furthermore, a multicenter study including 331 individuals with positive cultures between 2019 and 2022 demonstrated clear reported case peaks during mid-2020 and mid-2021, coinciding with the pandemic period and supporting the hypothesis that COVID-19-associated healthcare pressures may have amplified transmission and case detection [44].

Across the Eastern Mediterranean region, the COVID-19 period appears to have accelerated both recognition and hospital spread of *C. auris*. In Lebanon, half of the first outbreak patients had concurrent COVID-19, and subsequent case-control and genomic analyses linked acquisition to severe COVID-19 as one of the main factors [14,58]. Israel showed a similar pattern, with a 30-fold rise in 2021, 23% of cases occurring in patients with COVID-19, and most outbreaks beginning in ventilated COVID-19 ICU patients before spreading to other wards [13]. Jordan also reported its first case in a patient recently hospitalized for COVID-19 [37].

The Egyptian experience appears to represent a later stage of emergence. Early surveillance efforts failed to detect the organism despite extensive screening. Khairat et al. examined 400 *Candida* isolates over a two-year period and found no *C. auris* isolates, suggesting that the pathogen was either absent or circulating at extremely low levels before 2020 [61]. In contrast, studies conducted during and after the pandemic began to identify increasing numbers of isolates. Mahmoud et al. detected eight *C. auris* isolates from bloodstream infections and catheter-associated specimens collected during 2021–2022 [39]. Another study reported *C. auris* among candidemia cases during 2021–2023 [62]. Most notably, an 18-month investigation published in 2025 identified 120 *C. auris* isolates from a single tertiary-care hospital, indicating that the organism had become firmly established within Egyptian healthcare settings within only a few years [63]. This progression from complete absence in earlier surveillance studies to large numbers of isolates after 2020 strongly supports a recent emergence pattern.

Comparable post-pandemic amplification of *C. auris* has been documented in other regions, particularly North America and Europe. In the United States, national surveillance showed a marked increase in clinical and screening detections between 2019 and 2021, and a single-center study from New York found that the reported cases of colonization or infection rose significantly from 2019 to 2022, indicating sustained expansion during the COVID-19 era [66]. In Europe, the Italian outbreak pattern was similarly temporally linked to the pandemic. For instance, the first case in Liguria was detected in July 2019, *C. auris* was identified in a COVID-19 ICU in February 2020, and case numbers then increased throughout 2020 and 2021, with 361 cases reported across 17 healthcare facilities by December 2022 [67]. At the continental level, European surveillance also recorded a rise in reported cases from 335 in 2020 to 655 in 2021 across more reporting countries, further supporting pandemic-associated amplification of hospital transmission [68]. Further, a recent report documented a rapid post-COVID expansion of *C. auris* in China and showed, by phylogenetic analysis of 81 isolates, the circulation of clades I, II, and III across China, indicating that multiple lineages can expand efficiently after pandemic-related healthcare disruption [69]. This study is useful because it shows that post-COVID expansion of *C. auris* is not simply an increase in case counts, but a genomic and epidemiologic process characterized by sustained hospital transmission, multiple clusters, and expansion of more than one lineage.

**Figure 4:**
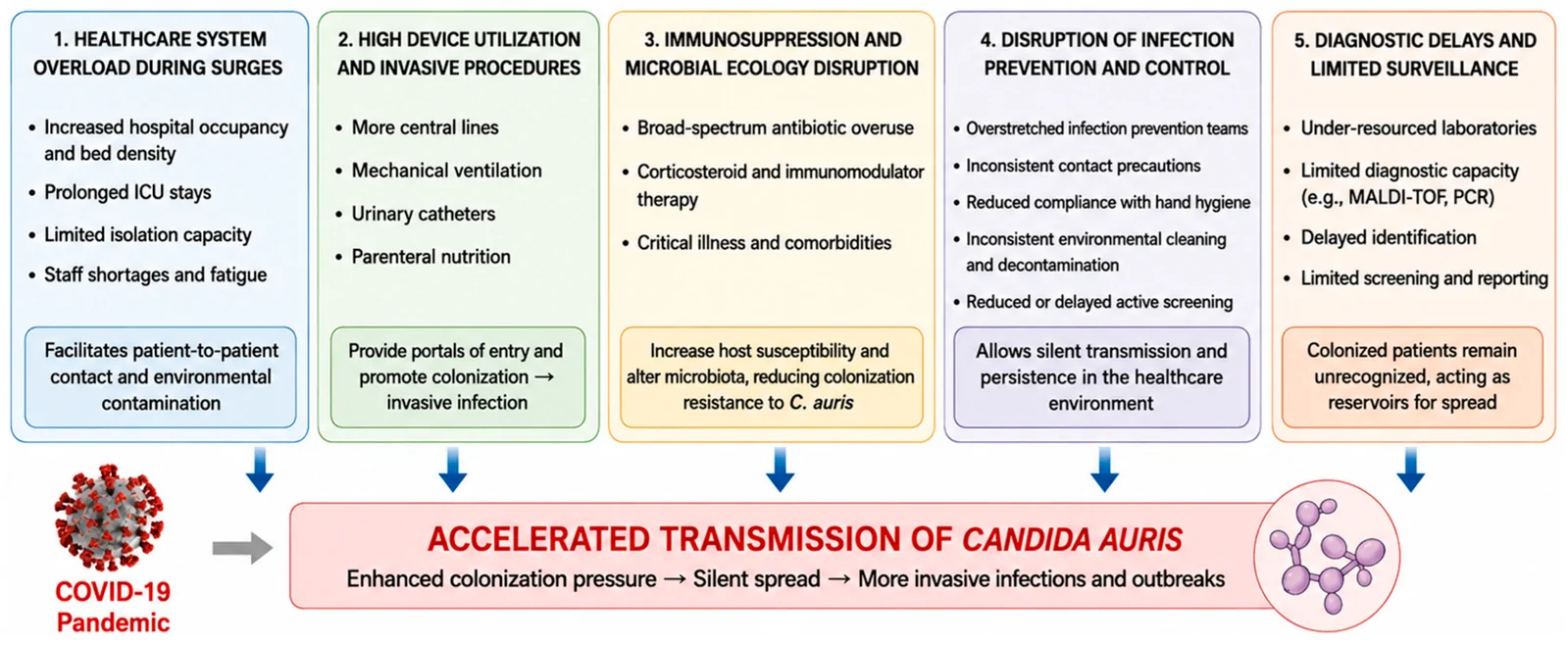
Multifactorial drivers of the Post-COVID-19 increase in *C. auris* cases and healthcare-associated transmission.

Taken together, these data support the inference that the COVID-19 pandemic acted as an accelerator of healthcare-associated *C. auris* dissemination through ICU crowding, high device utilization, and disruption of infection-prevention practices, rather than as the sole cause of emergence. The post-COVID rise in cases is most plausibly multifactorial. During pandemic surges, hospitals experienced increased occupancy, prolonged ICU stays, and more frequent use of invasive devices, all of which facilitate colonization and progression to invasive infection. In parallel, broad-spectrum antibiotic exposure and corticosteroid use likely increased host susceptibility and altered the microbial ecology of critically ill patients. Infection prevention and control systems were also under substantial strain, which may have reduced the consistency of active screening, environmental decontamination, and contact precautions, thereby allowing silent ward-level transmission to persist (Figure 4). Diagnostic delays may have further contributed, because under-resourced laboratories and limited surveillance can permit colonized patients to remain unrecognized while serving as reservoirs for ongoing spread. Accordingly, the apparent post-pandemic increase most likely reflects amplification of already established transmission pathways under extraordinary healthcare pressure, together with improved detection of cases that may previously have gone unnoticed.

### 4.3. Predominant Clade Distribution Across the Region

**Figure 5:**
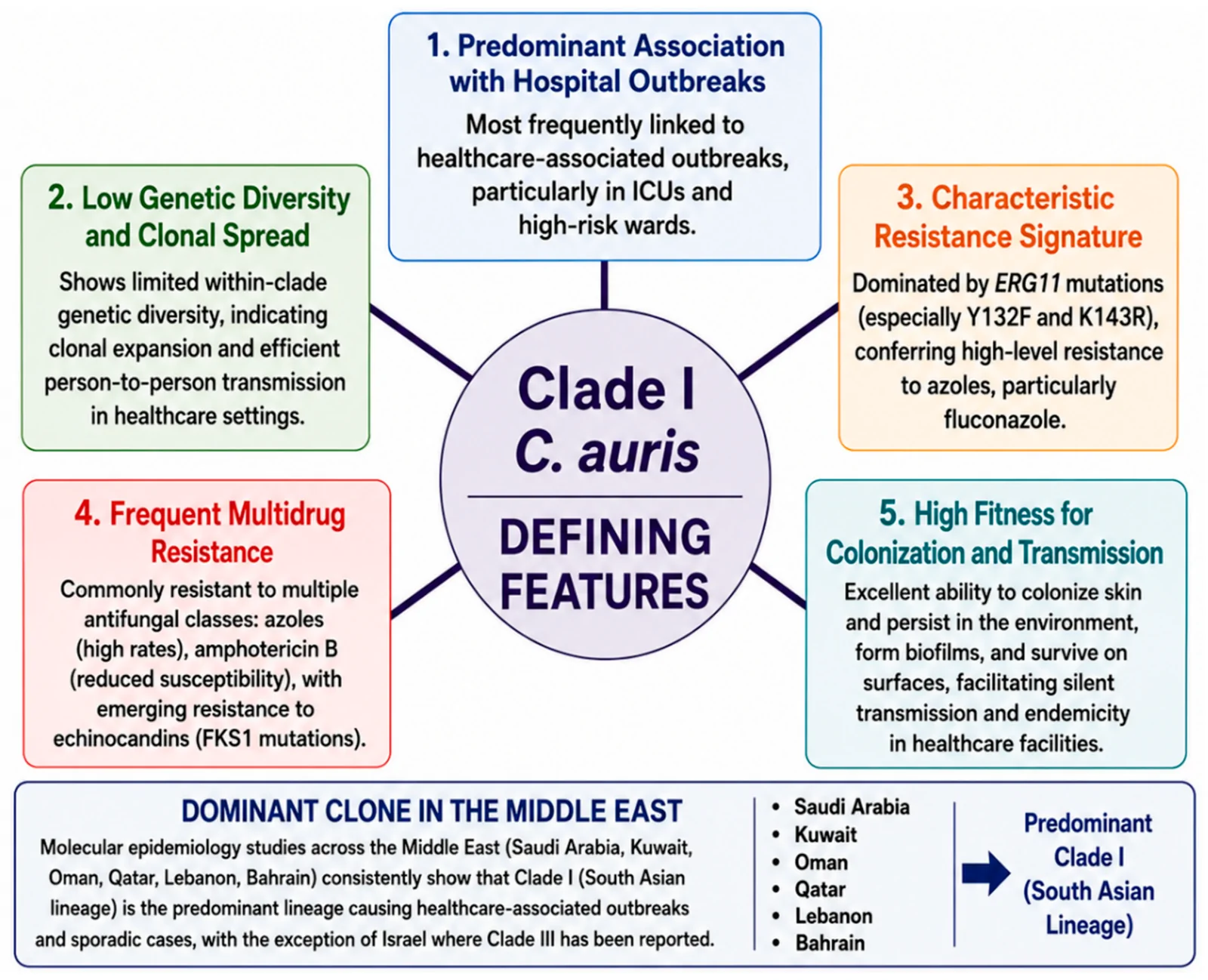
Defining features of *C. auris* Clade I (South Asian Lineage) and its predominance in the Middle East.

Across the Middle East, the available molecular evidence indicates a strong predominance of the South Asian lineage, clade I, across Saudi Arabia, Kuwait, Oman, Qatar, Lebanon, and likely Bahrain, where multiple studies using WGS, STR genotyping, ITS sequencing, and related approaches consistently clustered isolates within clade I and showed clonal nosocomial transmission (Figure 5). In Lebanon, all sequenced isolates from the COVID-19-associated outbreak belonged to clade I, with minimal genetic diversity, supporting sustained person-to-person spread within ICU settings [59]. Qatar and Saudi Arabia showed the same pattern, with both pre-pandemic and COVID-era outbreak isolates assigned to the South Asian lineage [19,43]. Kuwait likewise showed long-standing clade I dominance across serial isolates and outbreak investigations, including several reports of multidrug resistance and limited echinocandin escape [31,49,51,52]. Israel represents the principal regional exception, because early isolates were linked to South African strains and a clade III clone became predominant after 2021 during the COVID-19 hospitalization surge [13]. Egypt remains less informative at the clade level because the available studies did not report formal clade assignment, although the resistance-associated mutations described are compatible with clade I/III-associated lineages.

Clade I, also designated the South Asian lineage, is one of the five major geographically structured lineages of *C. auris* and was first recognized in South Asia, particularly India and Pakistan [10]. Population genomic studies have shown that clade I has spread across multiple continents and has been identified in numerous healthcare-associated outbreaks, highlighting its exceptional capacity for nosocomial transmission and persistence within healthcare environments [70]. Unlike clade II, which is predominantly associated with ear infections and has rarely been linked to large hospital outbreaks, clade I is frequently recovered from invasive infections including candidemia and has repeatedly been implicated in sustained healthcare-associated transmission events [70,71]. From a resistance perspective, clade I is notable for its high prevalence of azole resistance and its association with characteristic *ERG11* mutations, which contribute to reduced fluconazole susceptibility. Genomic analyses have further demonstrated that clade I contains a substantial proportion of multidrug-resistant isolates and includes strains resistant to all three major antifungal classes, emphasizing its clinical significance [72,73].

The predominance of clade I throughout the Middle East is therefore epidemiologically important because it suggests that the regional epidemic is largely driven by a lineage already recognized globally for efficient healthcare-associated spread, environmental persistence, outbreak potential, and antifungal resistance. The repeated identification of clade I in the Middle East indicates a remarkable degree of regional homogeneity compared with other parts of the world where multiple clades circulate simultaneously. This pattern may reflect both historical introduction events and successful adaptation of clade I to healthcare settings across the region.

Major defining features of *C. auris* Clade I (South Asian lineage) include its association with healthcare-associated outbreaks, low genetic diversity and clonal spread, characteristic azole-resistance mutations, frequent multidrug resistance, and enhanced colonization and transmission capacity. Molecular epidemiological studies indicate that Clade I is the predominant lineage reported across most Middle Eastern countries.

### 4.4. Antifungal Resistance Profile

The antifungal resistance profile of *C. auris* reported across the Middle East and North Africa was characterized by near-universal resistance to fluconazole, variable susceptibility to amphotericin B, and generally preserved activity of echinocandins [62,63]. Across Gulf countries including Saudi Arabia, Kuwait, Oman, Qatar, Bahrain, and UAE, fluconazole resistance consistently exceeded 90% in most studies and frequently reached 100% [18,40,43]. Amphotericin B resistance was more heterogeneous, ranging from occasional non-susceptibility in Saudi Arabia and Kuwait to very high resistance rates in Oman, Qatar, and the UAE [18,40]. In contrast, echinocandins largely retained activity against regional isolates, although the emergence of resistance mediated by *FKS1* hotspot mutations was reported in the UAE, Qatar and Kuwait, highlighting the potential for therapy-driven evolution of multidrug-resistant strains [34,42]. Molecular studies further demonstrated the widespread presence of resistance-associated *ERG11* mutations, particularly *Y132F* and *K143R*, which are known drivers of azole resistance and are commonly associated with the predominant South Asian clade [41].

Notably, although echinocandins generally remained active across most Middle Eastern isolates, the region has also documented the emergence of the most extreme resistance phenotypes. In the UAE, a national surveillance study identified a small but important proportion of isolates that were classified as pandrug-resistant, alongside additional extensively drug-resistant strains [45]. In Qatar, genomic analysis also documented an isolate resistant to three antifungal classes, indicating that resistance can progress beyond the usual fluconazole-dominant pattern when selective pressure is intense [42]. Importantly, the Middle East has also reported resistance at the most extreme end of the spectrum. In Egypt, a candidemia study found that all three *C. auris* isolates were resistant to every antifungal agent tested, including fluconazole, voriconazole, caspofungin, micafungin, amphotericin B, and flucytosine, representing a truly pan-resistant phenotype [62]. Further, Egyptian cases showed an uncommon resistance phenotype, where *C. auris* isolates were susceptible to voriconazole but non-susceptible to caspofungin and amphotericin B, indicating that echinocandin resistance may emerge even when azole activity is still retained [39].

This places the Middle Eastern profile in line with the broader global behavior of *C. auris*, which is often characterized by near-universal azole resistance, variable amphotericin B susceptibility, and initially preserved echinocandin activity, but with the recognized capacity to accumulate stepwise resistance to all major antifungal classes [74]. Thus, while the dominant regional phenotype remains azole-resistant and often echinocandin-susceptible, the presence of pandrug-resistant and multidrug-resistant isolates shows that the Middle East is not exempt from the highest-risk resistance trajectory described for *C. auris* worldwide.

### 4.5. Shift from Colonization to Invasive Disease

A central theme across the reviewed studies is that *C. auris* frequently presents first as colonization and then progresses to invasive disease in a subset of high-risk patients. This transition was most clearly demonstrated in Saudi Arabia, where a case–control study found that 44 of 334 colonized patients developed invasive infection, with progression associated with central line use, mechanical ventilation, parenteral nutrition, and recent abdominal surgery; mortality was also substantially higher in the infection group than in colonized patients [28]. A broader multicentric Saudi analysis reinforced this pattern, showing 291 infected and 220 colonized cases, with most cases occurring in the ICU and mortality numerically greater among infected patients [29]. Screening-based studies further showed that colonization often predominated at axilla, groin, and nasal sites, whereas bloodstream and other sterile-site isolates represented the invasive end of the spectrum [26,29].

The same colonization-to-infection gradient was evident in Qatar. In the multicenter Qatari cohort, 13.6% of individuals with a positive culture had invasive *C. auris* disease, and invasive cases were more likely to require ICU care, mechanical ventilation, and central venous catheters; mortality was markedly higher in invasive disease than in colonization [44]. Similar findings were reported in the bloodstream infection series, in which nearly all patients were critically ill, and the majority of fungemia episodes were central line-related [43]. In the UAE, most detections were hospital-acquired, and many were recovered from urine or surveillance specimens rather than from invasive sites, suggesting that silent carriage remained common before candidemia emerged [46]. Further, although the first reported UAE case did not include a documented pre-invasive colonization screen, it strongly illustrates the pathway by which *C. auris* can progress from healthcare-associated persistence to invasive candidemia [34]. The patient had prolonged hospitalization, recurrent ICU admissions, multiple prior infections, and ongoing catheter exposure, all of which are classic settings for *C. auris* acquisition and persistence. The organism was initially misidentified as *Candida haemulonii*, delaying definitive recognition, and candidemia recurred despite caspofungin therapy and catheter replacement. Subsequent isolates showed increasing MICs to amphotericin B, caspofungin, and voriconazole, indicating selective pressure during treatment. Taken together, this case supports the concept that *C. auris* may remain clinically unrecognized during colonization or early persistence and later emerge as invasive bloodstream infection in critically ill patients with prolonged healthcare exposure [34]. The clearest Middle East example comes from Qatar. In March 2020, a 55-year-old man admitted to the ICU for H1N1 pneumonia was found to have *C. auris* colonization of his respiratory tract and urinary catheter. Two weeks later, he developed *C. auris* candidemia. The arterial line was removed, and the patient received anidulafungin, to which the isolate was susceptible. The fungemia cleared within 4 days of therapy [43]. This case highlights that *C. auris* can persist silently on indwelling devices and mucosa before invasive disease.

Comparable patterns were also observed in Lebanon and Kuwait, where colonized patients formed an important reservoir for ongoing transmission and later invasive disease. It is obvious that these data support the concept that colonization is not a benign state in *C. auris* epidemiology but rather a key intermediate step in the pathway to invasive infection. The shift is favored by ICU exposure, invasive devices, prolonged hospitalization, and heavy healthcare contact, and it is accompanied by a clear increase in mortality.

To place these findings in a broader context, published data from other regions show a highly similar epidemiologic pattern. In the United States, the transition from colonization to invasive *C. auris* disease has been quantified more directly than in many other settings. A recent CDC analysis found that among 21,195 patients with documented *C. auris* colonization, 6.9% later had a positive clinical specimen and 2.8% developed bloodstream infection, showing that colonization is a meaningful precursor to invasive disease in a clinically important minority of patients [75]. This trend occurred in parallel with a steep national expansion in case detection, as U.S. clinical cases rose from 476 in 2019 to 1471 in 2021 and screening detections tripled from 2020 to 2021, indicating that both carriage and invasive disease increased during the same period [66]. European data show a broadly similar pattern, although the literature there more often reports combined “colonization or infection” counts rather than a single quantified progression rate from carriage to invasion. In the EU/EEA, reported *C. auris* colonization-or-infection cases increased from 335 in 2020 to 655 in 2021, and later ECDC surveillance found 4012 cases across 2013–2023, with 1346 cases reported in 2023 alone [68,76]. It can be indicated that the European experience matches the Middle Eastern pattern in showing that ICU exposure, prolonged hospitalization, and infection-control strain facilitate the movement from colonization to invasive disease, but Europe has more often been described at the level of outbreak expansion and mixed carriage/infection rather than explicit colonization-to-infection conversion rates.

### 4.6. Mortality Burden and Clinical Outcomes

Across the reviewed Middle Eastern cohorts, *C. auris*–associated mortality was consistently high and increased with invasive disease. In Saudi Arabia, 30-day all-cause mortality was 47.8% in a bloodstream-infection cohort [27], in-hospital mortality reached 65.9% among patients who progressed from colonization to invasive infection [28], and crude mortality was 41.5% in a multicentric outbreak analysis [29]. In Qatar, 30-day mortality was 37.8% in the invasive-disease cohort [44] and 41.6% in the bloodstream-infection cohort [43], whereas the UAE reported 27.5% overall mortality and 36.1% mortality among blood-culture isolates [45]. Comparable lethality was observed in Oman, where crude mortality ranged from 39.1% to 53.1% across cohorts [53,55], and in Bahrain, where mortality reached 44.1% in one tertiary-care series, whereas the higher mortality of 85.5% reported in a candidemia survey reflected all Candida species rather than *C. auris* specifically [38,56].

These regional estimates are broadly concordant with global literature. Overall mortality in patients with *C. auris* infection ranged from 29% to 62%, whereas 30-day crude mortality ranged from 23.4% to 66.7%; in some studies, 27–31% of deaths were directly attributable to *C. auris* [77]. The mortality burden was not uniform and appeared to vary with case mix, geographic setting, healthcare infrastructure, and the extent of multidrug resistance [77,78]. This variation is clinically important because *C. auris* commonly affects critically ill patients with prolonged ICU exposure, invasive devices, renal impairment, sepsis, and prior antifungal exposure, all of which amplify the risk of progression from colonization to invasive disease [77,78]. In parallel, the organism exhibits strong persistence in healthcare environments, frequent misidentification by routine phenotypic systems, and limited susceptibility to commonly used antifungals, thereby delaying definitive therapy and infection-control interventions [77,78].

Collectively, these data support the interpretation that the high mortality of *C. auris* is multifactorial and is driven predominantly by severe host vulnerability, delayed identification, treatment limitations, biofilm-associated persistence, and the capacity of the organism to cause outbreaks in strained healthcare settings rather than by infection alone.

### 4.7. Misidentification and Diagnostic Challenges

Misidentification is a defining diagnostic problem for *C. auris* and has repeatedly delayed recognition during outbreaks. Conventional phenotypic systems may report the organism as *C. haemulonii*, *C. duobushaemulonii*, *C. famata*, *C. lusitaniae*, *C. sake*, *or even Rhodotorula glutinis*, because *C. auris* is absent from, or poorly represented in, many commercial databases [79,80]. Primary studies have shown that this problem is not theoretical. Isolates initially labeled *as C. haemulonii* or *C. famata* by routine systems were later confirmed as *C. auris* by MALDI-TOF MS or sequencing, demonstrating that conventional workflows can substantially underestimate the true burden [81]. Accurate identification therefore depends on methods with curated databases or sequence-level resolution. MALDI-TOF MS is reliable when *C. auris* is included in the reference library, and confirmatory DNA sequencing of the D1–D2 or ITS regions remains a robust alternative when MALDI-TOF is unavailable; whole-genome sequencing is the most definitive approach for outbreak investigation and clade assignment. In contrast, for VITEK 2, the updated software library is an improvement, but it is not fully definitive for *C. auris*. CDC states that software version 8.01 includes *C. auris*, yet it should still be interpreted cautiously because it has limited ability to correctly identify some clades, and as of the current CDC guidance, it consistently identifies some, but not all, clades [82]. Therefore, any isolate reported as *C. auris*, *C. haemulonii*, or other members of the *C. haemulonii* complex should be confirmed by MALDI-TOF with an updated library or by DNA sequencing [79]. Despite these advances, major practical barriers remain, particularly in low- and Middle-income settings where MALDI-TOF, sequencing, and standardized antifungal susceptibility testing may be limited. Interpretation is further complicated by the lack of established *C. auris*-specific MIC breakpoints, so laboratories often rely on tentative CDC breakpoints or expert guidance rather than fully validated interpretive criteria.

Misidentification was repeatedly documented across the Middle East and represented a major barrier to timely recognition of *C. auris*. In Saudi Arabia, the first three cases were initially misidentified as *C. haemulonii* by the Vitek 2 system before confirmation by MALDI-TOF MS and whole-genome sequencing [16]. In Kuwait, an early series confirmed 158 *C. auris* isolates from 56 patients after many had first been reported as *C. haemulonii* by Vitek 2 [48]. A larger epidemiologic study showed that all 212 isolates recovered before 11 April 2018 were initially misidentified as *C. haemulonii* until the Vitek 2 Compact database was updated [49]. In Oman, the earliest report likewise noted that the isolates had been initially misidentified as *C. haemulonii* [33]. In the UAE, the first reported case was also misidentified by a conventional automated system as *C. haemulonii*, delaying recognition of persistent candidemia [34]. Taken together, these findings indicate that routine phenotypic platforms were insufficient for reliable species-level identification of *C. auris* in the region, particularly before database updates and access to MALDI-TOF MS or sequencing became available. This diagnostic limitation had direct clinical consequences because it delayed infection-control interventions, obscured outbreak recognition, and may have contributed to underestimation of the regional burden of *C. auris*.

### 4.8. Surveillance Approaches and Their Influence on Reported Trends

The observed trends in *C. auris* emergence across Middle Eastern and North African countries are based on heterogeneous surveillance approaches, which should be considered when comparing the reported burden between countries. National or multicenter surveillance systems provided the most comprehensive estimates in some settings. For example, Saudi Arabia implemented multicenter surveillance across several hospitals and documented a substantial increase in reported outbreaks during 2020–2022, while national surveillance in the United Arab Emirates identified a large number of non-duplicate isolates over the 2018–2021 period. In Qatar, multicenter hospital surveillance was complemented by outbreak investigations and molecular/genomic analyses, allowing temporal clustering of cases to be identified.

In contrast, several countries have primarily relied on hospital-based retrospective studies, candidemia surveillance, outbreak investigations, or targeted screening of high-risk populations. Active screening of intensive-care-unit patients has been particularly important for identifying colonization that would otherwise remain undetected, whereas candidemia-based surveillance captures predominantly invasive disease and therefore does not represent the overall colonization burden. Outbreak investigations may also produce short-term increases in reported cases because intensive screening is initiated after an index case or cluster is recognized. Molecular and genomic surveillance, where available, further improves the ability to distinguish persistent transmission from repeated independent introductions.

Accordingly, the apparent differences in the magnitude and temporal pattern of *C. auris* emergence between countries should not be interpreted solely as differences in the underlying epidemiological burden. Variation in surveillance intensity, population coverage, diagnostic capacity, screening strategies, healthcare-facility participation, and the type of surveillance data collected can substantially influence the number of cases identified and reported. Thus, countries with established national or multicenter surveillance systems may appear to have a higher burden partly because of more systematic case detection, whereas countries relying mainly on individual hospitals or outbreak reports may underestimate the extent of transmission. The reported trends presented in this review therefore reflect both the epidemiological emergence of *C. auris* and the evolution of surveillance and detection capacity across the region.

## 5. Conclusions

The evidence reviewed indicates that *C. auris* has become an established healthcare-associated fungal threat across multiple countries in the Middle East and North Africa, although the magnitude of the reported burden differs substantially between countries. The strongest evidence comes from the Gulf region, while the Eastern Mediterranean and North Africa show more heterogeneous and comparatively fragmented documentation, making direct comparisons of reported case numbers difficult. Across the region, recurrent healthcare-associated transmission, colonization of high-risk patients, invasive disease, diagnostic misidentification, and substantial antifungal resistance represent interconnected challenges requiring coordinated surveillance and infection-prevention responses.

### 5.1. Actionable Regional Recommendations

#### 5.1.1. Surveillance

Countries should strengthen standardized national and hospital-based surveillance systems that capture both colonization and clinical infection, with harmonized case definitions and reporting variables to permit meaningful comparison across countries and over time. Active screening should be prioritized for patients at increased risk of colonization, particularly those with epidemiologic links to known cases, prolonged healthcare exposure, or complex and invasive medical care. Confirmed cases should be rapidly communicated to infection-prevention teams and relevant public-health authorities, and *C. auris* status should accompany patients during interfacility transfer to reduce the risk of silent dissemination between healthcare facilities. Regional surveillance should also incorporate molecular typing or whole-genome sequencing where feasible to distinguish independent introductions from healthcare-associated transmission and to monitor the emergence and dissemination of resistant lineages [83].

#### 5.1.2. Diagnostics

Diagnostic laboratories should ensure that their yeast-identification platforms have validated and updated databases capable of recognizing *C. auris*, while suspected isolates that cannot be reliably identified should be referred to reference laboratories for confirmation. MALDI-TOF MS and molecular methods, including PCR and sequencing, should be incorporated into regional diagnostic networks where available because conventional phenotypic systems have historically produced clinically important misidentifications. Laboratories should establish referral pathways that allow rapid confirmation of suspected isolates and access to antifungal susceptibility testing, particularly when resistance or treatment failure is suspected.

#### 5.1.3. Antifungal Stewardship

Antifungal stewardship programs should integrate species-level identification, susceptibility testing, clinical assessment, and regular review of antifungal therapy to reduce unnecessary exposure and support appropriate treatment selection. Because fluconazole resistance is widespread and resistance to echinocandins and other antifungal classes has emerged in the region, treatment decisions should not rely on assumptions based solely on geographic origin or historical susceptibility patterns. Routine antifungal susceptibility testing and investigation of unusual susceptibility profiles should therefore be strengthened, particularly for invasive infections and patients who fail initial therapy.

## 6. Limitation

A major limitation is that much of the regional evidence is derived from retrospective single-center studies, outbreak reports, case series, and targeted surveillance rather than nationally representative prospective surveillance, which limits direct comparison of reported case numbers and mortality between countries. Further, the apparent lower burden in several North African and Eastern Mediterranean countries may therefore partly reflect differences in case detection, laboratory capacity, surveillance intensity, and publication activity rather than a genuinely lower prevalence of *C. auris*. Finally, mortality estimates should also be interpreted cautiously because many cohorts consisted of critically ill patients with substantial comorbidity, and the studies used different definitions of mortality and different follow-up periods. Accordingly, reported mortality should not automatically be interpreted as *C. auris*-attributable mortality unless the original study specifically established causality.

## Figures and Tables

**Table 2:** Major *C. auris* outbreaks reported in the Middle East.

Country	Occurrence Date/Period	Major Outbreak or Series	Cases	Mortality Rate	Reference
Saudi Arabia	March 2018–June 2019	First tertiary-care hospital outbreak	34	20.6%	[18]
Saudi Arabia	2020	ICU outbreak	7	85.7%	[20]
Saudi Arabia	2020–2022	National multicenter outbreak overview	511 cases; 186 outbreaks	41.5% crude mortality	[29]
Qatar	October 2018–August 2019	First hospital outbreak	13	60%	[35]
Qatar	2020–2021	COVID-19 period	76	Not reported	[42]
Kuwait	2018	High-dependency unit outbreak	71	50% crude mortality	[52]
Oman	April 2018–April 2019	Sohar outbreak	32	53.1% crude 30-day mortality	[53]
Bahrain	Late 2021–2022	Salmaniya Medical Complex outbreak	59	44.1% crude mortality	[38]
Lebanon	October 2020 onward	ICU outbreak	14	Not reported	[14]

## Data Availability

No new data were created or analyzed in this study. Data sharing is not applicable to this article.
